# Improvement in Yield of Extracellular Vesicles Derived from Edelweiss Callus Treated with LED Light and Enhancement of Skin Anti-Aging Indicators

**DOI:** 10.3390/cimb45120634

**Published:** 2023-12-16

**Authors:** Mi-Jung Kim, Hoon Ko, Ji-Young Kim, Hye-Jin Kim, Hwi-Yeob Kim, Hang-Eui Cho, Hyun-Dae Cho, Won-Sang Seo, Hee-Cheol Kang

**Affiliations:** 1 Human & Microbiome Communicating Laboratory, GFC Co., Ltd., Hwaseong 18471, Republic of Korea; mj2.kim@gfcos.co.kr (M.-J.K.); jy.kim@gfcos.co.kr (J.-Y.K.); hy.kim@gfcos.co.kr (H.-J.K.); 2 Creative Innovation Research Center, Cosmecca Korea Co., Ltd., Seongnam 13488, Republic of Korea; kohoon329@cosmecca.com (H.K.); kimhy0119@cosmecca.com (H.-Y.K.); bioche1975@cosmecca.com (H.-E.C.); color@cosmecca.com (H.-D.C.)

**Keywords:** *Leontopodium alpinum* L., extracellular vesicles, plant-derived EVs, LED, magenta

## Abstract

The process of skin aging is currently recognized as a disease, and extracellular vesicles (EVs) are being used to care for it. While various EVs are present in the market, there is a growing need for research on improving skin conditions through microbial and plant-derived EVs. Edelweiss is a medicinal plant and is currently an endangered species. Callus culture is a method used to protect rare medicinal plants, and recently, research on EVs using callus culture has been underway. In this study, the researchers used LED light to increase the productivity of Edelweiss EVs and confirmed that productivity was enhanced by LED exposure. Additionally, improvements in skin anti-aging indicators were observed. Notably, M-LED significantly elevated callus fresh and dry weight, with a DW/FW ratio of 4.11%, indicating enhanced proliferation. Furthermore, M-LED boosted secondary metabolite production, including a 20% increase in total flavonoids and phenolics. The study explores the influence of M-LED on EV production, revealing a 2.6-fold increase in concentration compared to darkness. This effect is consistent across different plant species (*Centella asiatica, Panax ginseng*), demonstrating the universality of the phenomenon. M-LED-treated EVs exhibit a concentration-dependent inhibition of reactive oxygen species (ROS) production, surpassing dark-cultured EVs. Extracellular melanin content analysis reveals M-LED-cultured EVs’ efficacy in reducing melanin production. Additionally, the expression of key skin proteins (FLG, AQP3, COL1) is significantly higher in fibroblasts treated with M-LED-cultured EVs. These results are expected to provide valuable insights into research on improving the productivity of plant-derived EVs and enhancing skin treatment using plant-derived EVs.

## 1. Introduction

Serving as the body’s largest defensive barrier persistently exposed to external elements, the skin is susceptible to heightened vulnerability, making it prone to injuries influenced by a range of factors, such as genetic predisposition, lifestyle choices, nutritional habits, solar radiation exposure, and diverse environmental factors [1,2]. The aging process and skin diseases create a complex burden that extends beyond individual impacts, affecting mental, social, and financial aspects. Despite global attention focused on understanding and addressing skin abnormalities, current treatments fall short of desired outcomes. Hence, there is an urgent call for additional research to develop more effective therapeutic strategies for skin-related conditions [3,4]. Recently, extracellular vesicles (EVs) have garnered attention as a potential approach to address skin aging [5].

EVs exhibit favorable biological characteristics for the treatment of skin conditions. These EVs are small lipid bilayer nanoparticles released by various cell types into the extracellular space. They can be categorized into three types based on size and biogenesis: exosomes (50–200 nm), microvesicles (MVs) (100–1000 nm), and apoptotic bodies (500–5000 nm) [6,7,8]. Exosomes are generated through the endosomal pathway. EVs possess qualities that hold promise as a method for protecting skin against the aging process. They naturally carry a range of biomolecules, including proteins, lipids, DNA, and RNA, essential for intercellular communication and modulating molecular activities in recipient cells [9,10].

Both mammalian and plant-derived EVs have shown therapeutic potential in improving skin conditions, such as wound healing, reducing pigmentation, minimizing wrinkles, and preventing scar formation [11,12,13,14]. Microbial EVs separated from the human skin also have anti-aging effects on the skin [8]. Plant EVs were first identified in the 1960s, but our understanding of them remains limited. A recent research investigation suggests that plant EVs have crucial functions in regulating their immune system, engaging in defense mechanisms, promoting symbiotic relationships between plants and microbes, and facilitating communication across cells and different biological kingdoms. This is achieved through the transportation of RNAs, proteins, and bioactive compounds [15,16]. Exploring the application of plant EVs in the realm of skin conditions, research has revealed that, similar to mammalian EVs, they contribute to the rejuvenation of the skin [17]. However, an increase in EVs due to contact with pathogens is not desired in the market for safety and aesthetic reasons. For this reason, alternative methods to increase EVs are needed. Enhancing the production of EVs can be achieved not only by utilizing the mentioned pathogens but also through various factors such as chemicals, oxygen levels, pH, radiation, and culture methods [18]. Among these factors, our focus lies on the use of light, a form of radiation, for promoting EV production.

Light is vital for plant growth, playing a key role in photosynthesis and energy generation. While intense light enhances photosynthesis and growth, excessive exposure can harm tissues. Optimizing photosynthetic efficiency requires providing plants with high-quality light tailored to specific wavelength ranges [19]. Light-emitting diodes (LEDs) serve as an artificial light source with a tailored spectrum to meet the specific growth needs of plants. LEDs offer advantages such as low energy consumption, minimal heat emission, and a long lifespan [20]. The impact of LEDs on in vitro-cultured plantlets has been reported in studies involving potato [21], Cymbidium [22], and strawberry [23]. However, excluding a few cases, there are not many instances of cultivating plant callus using LED lighting [24,25]. Moreover, the focus is often on specific phytochemical production [24,25]. Reports related to LEDs and calluses have been confirmed for plants such as *Hyoscyamus reticulatus* [26] and *Ruta chalepensis* [27]. Nevertheless, the emphasis is on the specific enhancement of components through LED illumination in plant callus. Notably, there is currently no reported increase in plant EVs due to LED exposure.

Edelweiss, a perennial Alpine flower (*Leontopodium alpinum* L.), has a long history of use in traditional medicine to alleviate abdominal aches, bronchitis, diarrhea, dysentery, and fever [28,29]. In recent times, a number of studies have demonstrated the effectiveness of Edelweiss extracts in reducing inflammation in both mice and rats [28], as well as in human keratinocytes and endothelial cells [29]. Edelweiss also contains antioxidants, including leontopodic acid A and 3,5-dicaffeoylquinic acid, which can serve as anti-aging agents [30]. However, given the protected and endangered status of the Edelweiss plant [31], the advancement of Edelweiss cell tissue culture technology is of paramount significance in the production of Edelweiss as a primary raw material for cosmetics. As a result of its challenging natural habitat, limited resources, and growing human demand, the species is now classified as rare. Consequently, the commercial availability of this plant for industrial applications is being promoted through the utilization of cell tissue culture technology aimed at regenerating Edelweiss plants and safeguarding endangered species [32]. Research results confirm the composition of Edelweiss cultured as simple callus, but LED was not applied, and, furthermore, EV production was not studied [33].

The aim of our study is to establish suitable LED application conditions for Edelweiss callus and investigate whether key indicators are related to growth increases. Specifically, we seek to confirm a trend of increased EVs through suitable LED exposure and assess enhancements in skin-related indicators, such as antioxidant properties, skin whitening, skin regeneration, skin elasticity, moisturization, and skin barrier function. As of now, there are no reported instances of EV generation through LED exposure.

## 2. Materials and Methods

### 2.1. Sterilization of Plant Material and Seeds

*Leontopodium alpinum* L. (Edelweiss) seeds were procured from Chiltern Seeds (Wallingford, UK). The seeds underwent a comprehensive sterilization procedure, commencing with a 5 min wash in tap water, followed by a 1 min immersion in 95% (*v*/*v*) ethanol. Subsequently, the seeds were submerged in a 50% (*v*/*v*) commercial bleach solution containing 0.05% detergent Tween-20 for 20 min. They were then rinsed three to four times with sterile distilled water under a laminar flow cabinet to ensure thorough sterilization. For germination, these sterile L. alpinum seeds were transferred to hormone-free solid MS media and incubated in darkness for three days at 25 °C [34]. After this dark incubation period, they were relocated to a phytotron under a photoperiod of 16 h of light and 8 h of darkness at 25 °C. *Centella asiatica* L. (Cica), acquired from Centella Farm (Hapcheon, Republic of Korea), underwent in vitro cultivation using the leaf sterilization method. *Panax ginseng* (Ginseng), supplied from the fields of the National Institute of Horticultural and Herbal Science, Rural Development Administration (Eumseong, Republic of Korea), underwent in vitro cultivation using the root sterilization method.

### 2.2. Callus Induction

To initiate the development of callus in Edelweiss, segments of stem and leaf measuring 0.5 to 1 cm, devoid of nodules, were extracted from 2 to 3-week-old seedlings. These explants were then positioned on a medium enriched with MS, 3% (*w*/*v*) sucrose, and 0.4% (*w*/*v*) gelatin. The medium also featured a blend of 0.5 mg/L benzyl adenine (BA) and 0.3 mg/L 2,4-dichlorophenoxyacetic acid (2,4-D). For the induction of callus in *Centella asiatica*, stem and leaf explants of 0.5 to 1 cm, without nodules, were obtained from sterilized leaves. These explants were placed on an MS medium containing 3% (*w*/*v*) sucrose and 0.4% (*w*/*v*) gelatin, along with 1 mg/L 2,4-dichlorophenoxyacetic acid (2,4-D). To prompt callus formation in *Panax ginseng*, root explants of 0.5 to 1 cm were derived from sterilized ginseng roots. These root explants were positioned on an MS medium supplemented with 3% (*w*/*v*) sucrose, 0.4% (*w*/*v*) gelatin, and 2 mg/L indole-3-butyric acid (IBA). The incubation was carried out in darkness at 25 °C. Subsequently, calli emerged from the explants and were subcultured every 4 weeks to establish a uniform line on an MS medium with consistent supplementation of plant growth regulators.

### 2.3. Spectral Light Treatments

**Figure 1:**
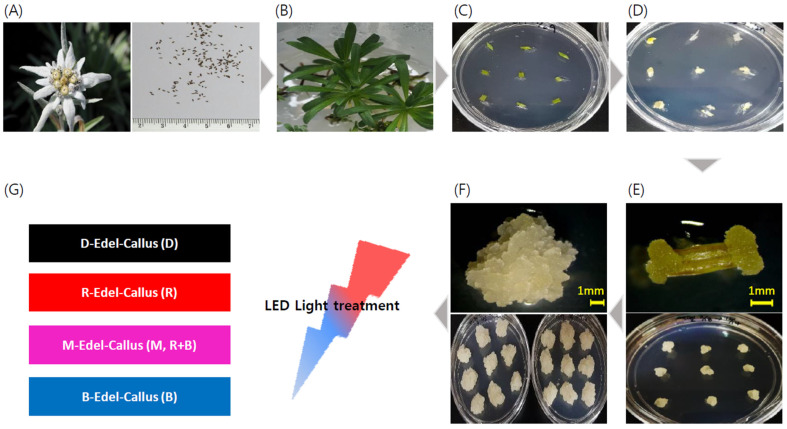
Flowchart for the Edelweiss callus induction process and illumination conditions. (**A**) Sourcing Edelweiss flowers and seeds. (**B**) Cultivating Edelweiss plants in vitro from sterilized seeds. (**C**) Preparing explants from Edelweiss leaves for callus induction. (**D**) Initiating callus formation from leaf explants. (**E**) Promoting callus induction and ensuring the uniformity of callus through subculturing. (**F**) Achieving homogeneous Edelweiss callus. (**G**) Administering LED light treatment to the uniform callus cultures.

High-quality cell lines, along with approximately 3 mm diameter callus clusters, were subcultured for experimentation. Various LED treatments were employed as physical stimuli to facilitate callus development: red LEDs (635 nm) for 24 h, denoted ‘R’; blue LEDs (448 nm) for 24 h, denoted ‘B’; magenta LEDs (a 50% red and 50% blue mix) for 24 h, labeled ‘M’; and a 24 h period of darkness as the control, labeled ‘D’. The callus was maintained in a controlled culture room at a temperature of 25 ± 2 °C, with light intensity ranging from 40 to 50 μ mol/m^2^s, measured by a Lux meter (SU10, Jeiotech, Daejeon, Korea) within the growth chamber. After four weeks of cultivation, visual observations were conducted to assess morphological differences among the callus, followed by harvesting for subsequent extraction and analysis (Figure 1).

### 2.4. Establishment of Callus Suspension Culture

The creation of a callus suspension culture involved the transfer of 9 g of white and friable callus into 1000 mL flasks, each containing 300 mL of fresh MS liquid medium at a seeding rate of 3%. This medium was enriched with BA (0.5 mg/L), 2,4-D (0.3 mg/L), and sucrose (30 g/L), and it omitted gelatin, maintaining a pH of 5.8. The culture flasks were placed on a horizontal shaker and agitated at 120 rpm within a temperature-controlled environment of 25 ± 2 °C. These cultures were subjected to various LED treatments for a duration of 4 weeks.

### 2.5. Preparation of Callus Extracts

The induced callus was nurtured and cultivated on MS basal medium enriched with a combination of 0.5 mg/L BA and 0.3 mg/L 2,4-D under three distinct LED lighting conditions (red, blue, magenta) or kept in darkness (control). Following 30 days of cultivation, the callus was carefully harvested, separated from the medium, and subjected to drying at a temperature range of 50~60 °C.

Subsequently, each dried callus was finely ground, and 1 g of the resulting powder was extracted using a hot-water method with 100 mL of water for analysis of total phenolic compounds and total flavonoid compound analysis [35], achieving a final concentration of 1%. After hot-water extraction, centrifugation was performed at 4000 rpm for 10 min, followed by removal of the pellet. The supernatant was then filtered through a 0.22 μm syringe filter.

### 2.6. Analysis of Total Phenolic Compounds (TPC) and Total Flavonoid Compounds (TFC)

The determination of TPC was carried out following a modified method adapted for microplate assays [36]. In summary, 100 μL of Folin-Ciocalteu’s reagent (diluted fourfold) was added to 20 μL of the extract solution or gallic acid standards (ranging from 0 to 200 μg/mL). After a 5 min incubation, 75 μL of Na_2_CO_3_ was introduced, followed by a 2 h incubation at room temperature in the absence of light. The absorbance of the solution was measured at 750 nm using a Bio Tek 800TS spectrophotometer. This determination was performed in triplicate. The total polyphenol content of the extracts was expressed as mg of gallic acid equivalents (GAE) per g of dry weight (DW), calculated using the equation derived from the calibration curve [37].

The TFC analysis followed a protocol outlined by Sanaka with slight modifications [38]. In brief, solutions of 5% NaNO_2_, 10% AlCl_3_·6H_2_O, and 1N NaOH were prepared separately. Subsequently, 250 μL of the sample or catechin standards (ranging from 0 to 200 μg/mL) were combined with 1 mL of distilled water, and then 75 μL of 5% NaNO_2_ and 150 μL of 10% AlCl_3_·6H_2_O were added. The mixture was allowed to stand for 6 min. Following the addition of 500 μL of 1N NaOH, the reaction continued for 11 min, after which the absorbance was measured at 500 nm using a microplate reader. The total flavonoid content of the extracts was expressed as mg of catechin equivalents (CE) per g of dry weight (DW), calculated using the equation derived from the calibration curve.

### 2.7. High-Performance Liquid Chromatography (HPLC) Analysis

HPLC analysis was performed on 1% hot water extracts derived from callus cultures under three distinct LED conditions, and the control culture kept in darkness. An INNO 4.6 × 25 mm, 0.5 μm C18 column (Youngjin Biochrom, Seongnam, Gyunggi, Korea) was utilized for the analysis. The mobile phase consisted of two solvents: solvent A (0.3% Trifluoroacetic acid, TFA in deionized water) and solvent B (Acetonitrile), and the analysis was conducted over a 40 min period. The injection volume was set at 1 μL, and the flow rate was maintained at 0.8 mL/min.

### 2.8. Protein Extraction and Quantification

Total protein extraction from callus cultures grown under distinct LED conditions and a dark control involved grinding each callus with liquid nitrogen. The crude protein was extracted using a cell lysis buffer, including a proteinase inhibitor cocktail, for stability. Centrifugation at 4 °C, 15,000 rpm for 30 min facilitated protein elution into the buffer, with the supernatant collected.

Protein quantification utilized the bicinchoninic acid (BCA) method, involving the reaction of 20 μL of the sample with 180 μL of the BCA reagent in a 96-well plate for 1 h at 37 °C. Absorbance was measured at 562 nm with a microplate reader, and protein concentration was determined by comparing results to a bovine serum albumin (BSA) standard curve.

Additionally, total protein from callus cultures under different LED conditions and the dark control underwent electrophoresis on a 12% SDS/PAGE gel. This process allowed identification of protein patterns based on molecular weights, visualized through Coomassie staining.

### 2.9. EV Isolation and Analysis

For the isolation and analysis of EVs, a liquid suspension culture was performed. Following a four-week culture period, separation of callus and cultured media was achieved using 100-mesh and 300-mesh filters, followed by centrifugation of the media at 3000~4000× *g* for 20 min with a centrifuge. Only the supernatant was collected to eliminate cell residues, and this step was repeated for thorough removal of impurities.

The supernatant underwent centrifugation at 100,000 to 150,000× *g* for 2 h using an ultra-high-speed centrifuge, leading to the settling of EVs in the pellet layer. These EVs were then reconstituted in sterile water and filtered through a 0.22 μm syringe filter for sterilization. Finally, the acquired EVs from Edelweiss Callus were subjected to analysis using NTA equipment (ZetaView, Particle Metrix, Inning am Ammersee, Germany) to determine the size and concentration of each EV based on the culture treatment conditions. EVs derived from *Centella asiatica* and *Panax ginseng* were also cultured in callus using the same criteria as Edelweiss and were separated and obtained using the method described above.

### 2.10. Cell Viability

To assess the cell-based effectiveness of EVs, toxicity experiments were firstly conducted on various cell types. HaCaT cells (human keratinocytes), Detroit cells (human fibroblasts), and B16F10 cells (mouse-derived melanoma cells) were cultured in DMEM medium containing 10% FBS (Fetal Bovine Serum) and 1% penicillin/streptomycin (GIBCO, 10,000 U/mL) in an incubator at 37 °C with 5% CO_2_ for 24 h. The cultured cell lines were then plated in 96-well plates at a concentration of 0.5 × 10^4^ cells per well and cultured under the same conditions until they adhered to the plates. Afterward, purified water samples and EV samples matched with the target particles were injected and processed. After 24 or 48 h of treatment, the culture medium was removed, and a 10% WST-1 reagent was added to each well. The mixture was incubated for 2 h, and the absorbance was then measured at 450 nm using a BioTek Microplate Readers (Agilent, Santa Clara, CA, USA).

### 2.11. Measurement of Intracellular ROS Levels

To ascertain the oxidative effects of EVs, the H2DCF-DA experiment was conducted using HaCaT cells. HaCaT cells were seeded in a 96-well plate at a density of 1 × 10^4^ cells per well. They were then cultured in a 37 °C environment with 5% carbon dioxide for 24 h, allowing the cells to adhere. Subsequently, the culture medium was replaced with serum-free media for 24 h.

Following a 30 min incubation at 37 °C with 5mCM-H2DCFDA, we injected and processed purified water samples and EV samples tailored to the target particles. Additionally, a positive control using the appropriate concentration of Trolox was included. To induce the production of reactive oxygen species (ROS), the cells were exposed to 500 mJ/cm^2^ UVB radiation. Following UVB exposure, the cells were washed once with PBS, and then a culture solution containing EVs was added. Upon completion of the reaction, the fluorescence intensity was measured at excitation and emission wavelengths of 488 nm and 528 nm, respectively.

### 2.12. Measurement of Extracellular Melanin Production

An experiment to evaluate the whitening effects of EVs was conducted using B16F10 melanoma cells. The cells were seeded in 24-well plates at a concentration of 3 × 10^4^ cells per well using DMEM medium and cultured for 24 h at 37 °C with 5% CO_2_. To induce melanin production in the cells cultured for 24 h, α-MSH at a concentration of 50 nM was added. EVs at various particles were mixed with the medium and applied to each well. The cultures were maintained at 37 °C with 5% CO_2_ for 72 h, after which the culture solution was collected.

To measure the amount of extracellular melanin produced over the 72 h culture period, 1 mL of culture solution was taken, and the absorbance was recorded at 450 nm using an ELISA plate reader. The melanin production inhibition rate was calculated based on the absorbance of the blank, which used DPBS in place of the sample solution. A positive control using 200 ug/mL of β-arbutin was also included.

### 2.13. Western Blot Analysis

Detroit cells (fibroblasts) were treated with each EVs for 24 h. The cells were harvested, and intracellular total proteins were extracted using 1X RIPA buffer, which included a protease inhibitor cocktail (#4693116001, Roche, Basel, Switzerland) and 1 mM Phenyl-methanesulfonyl fluoride (PMSF). To denature the proteins, they were boiled at 100 °C for 5 min, separated by SDS-PAGE (12%) gel, and subsequently transferred onto nitrocellulose membranes (Bio-Rad, Hercules, USA) at 24 V and 50 W for 35 min.

The membrane was then blocked with 5% (*w*/*v*) skimmed milk in tris buffer solution tween (TBST) [0.1% Tween-20 in tris buffer solution (TBS)] for 1 h at room temperature. It was washed three times in TBS (10 mM Tris HCl, pH 8.0, 150 mM NaCl) for 10 min each and then incubated with primary antibodies, including Rabbit Anti-Human FLG (Fil-aggrin, #abx100249, Abbexa, Cambridge, UK), AQP3 (Aguaporin3, #ab307969, Abcam, Cambridge, UK), and COL1 (Collagen I, #ab255809, Abcam, Cambridge, UK). GAPDH (#ab181602, Abcam, Cambridge, UK) was used as a control. The secondary antibodies used were Goat anti-rabbit IgG-HRP (Santa Cruz, Dallas, USA) and Donkey anti-goat IgG-HRP (Santa Cruz, Dallas, USA) for 1 h. After washing with a TBST buffer, the mem-brane was developed using an ECL solution kit (Amersham, Buckinghamshire, UK) and subsequently analyzed. PageRuler™ Prestained Protein Ladder, 10 to 180 kDa (#26616, Thermo Scientific, Waltham, USA), was employed to verify protein sizes.

### 2.14. Statistical Analysis

The experiments were conducted in triplicate. Significance was determined using ordinary one-way ANOVA followed by the Student’s *t*-test, with a threshold of *p* ≤ 0.05 considered statistically significant. All reported data represent the mean ± standard error (SE) unless stated otherwise.

## 3. Results

### 3.1. Effects of LEDs on Edelweiss Callus Growth

There are reports on the simple callus culture and component analysis of Edelweiss, but research on investigating LEDs for the increase in bioactive substances and enhanced EV production is lacking [33]. Our study findings are derived from the observation that the application of specific wavelengths to *Panax vietnamensis* significantly enhanced callus growth and influenced ginsenoside production, particularly under specific LED conditions [25]. Edelweiss seeds underwent sterilization for in vitro culture and were germinated and grown under a 16 h light/8 h dark cycle. Callus formation was induced from leaf explants. Confirmation and selection of homogeneous cell lines were conducted through three to four rounds of step-culturing every four weeks (Figure 1).

**Figure 2:**
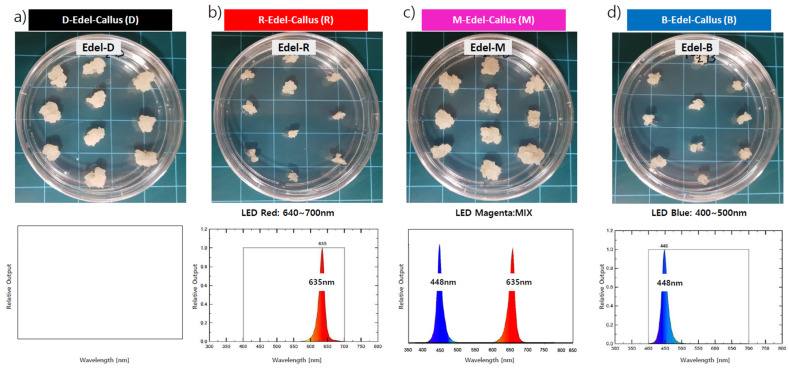
Following a 4-week cultivation period, we scrutinized the structure of Edelweiss callus cultures under four distinct lighting conditions. Specifically, selected Edelweiss callus lines were exposed to the following lighting environments: R-Edel-Callus (R)—red LED treatment (24 h, 635 nm), B-Edel-Callus (B)—blue LED treatment (24 h, 448 nm), M-Edel-Callus (M)—magenta LED treatment (24 h, comprising a 50% blend of red and blue), and D-Edel-Callus (D)—the control condition maintained in darkness for 24 h.

In the examination of the influence of red (R), blue (B), and magenta (M) LEDs on Edelweiss callus growth, high-performing cell lines were subcultured in ten spots on Petri dishes (approximately 3 mm in diameter). These cultures underwent exposure to three distinct LED conditions and a dark control (D) for a duration of four weeks at 25 °C ± 2 °C. The most notable callus proliferation occurred under the M-LED condition, demonstrating a growth rate comparable to the dark condition (D). Conversely, callus growth was slower under R-LED and B-LED conditions (Figure 2).

During the study, it was observed that the magenta light treatment resulted in the highest fresh callus weight, while the red-light treatment yielded the lowest. The ratio of dry weight to fresh weight (DW/FW) showed slight variations among the treatments, with magenta light having the highest ratio. To assess biomass differences in callus cultures under various light conditions, measurements were taken for fresh weight (FW), dry weight (DW), and the DW/FW ratio (%). The results revealed that the M-LED condition significantly increased callus fresh weight (8.76 g) and dry weight (0.36 g), resulting in a DW/FW ratio of 4.11% (Table 1). These findings suggest that M-LEDs contribute to enhanced callus proliferation compared to darkness. All values are averaged from five repetitions and presented in Table 1.

### 3.2. Comparison of Secondary Metabolites

In plant callus cultures with LED lighting, seldom utilized wavelengths demonstrate unique effects. In the case of *Ocimum basilicum*, blue light increases the total phenolic content, red light enhances total flavonoids, and blue light is effective for Rosmarinic acid and Eugenol. Continuous white light is found to be optimal for chicoric acid, while red light yields the highest anthocyanin content [24]. EVs derived from apples contain flavonoids and furanocoumarins known for their toxicity against fungal species and insects [39]. Flavonoids are produced within plant EVs to defend against external invasions. Flavonoids not only resist external pathogens but also provide beneficial effects to the skin.

The study analyzed total flavonoid content (TFC) and total phenolic content (TPC) using extracts from callus cultures grown under different conditions. Four separate callus extracts were prepared with catechin and gallic acid, serving as standards for TFC and TPC, respectively.

**Figure 3:**
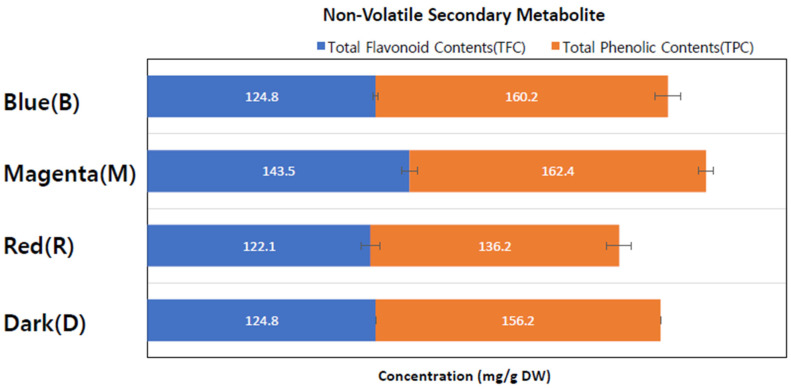
Analysis of non-volatile secondary metabolites in Edelweiss callus extracts cultured under varying LED light conditions. The Edelweiss callus extracts from different growth conditions were assessed for their total flavonoid content (TFC) and total phenolic content (TPC).

As indicated in Table 2 and Figure 3, the M-LED-treated callus extract exhibited the highest TFC content among all four extract conditions, measuring 143.5 ± 5.2 mg/g. The TFC contents in the other light conditions ranged from 122 to 125 mg/g, which were 14.8% to 17.6% lower. Concerning total phenolic contents, M-LED yielded the highest value at 162.4 ± 7.2 mg/g, followed by B-LED with 160.2 ± 2.2 mg/g, D with 156.2 ± 6.7 mg/g, and R-LED with 136.2 ± 4.2 mg/g.

In summary, the M-LED condition resulted in 305.9 ± 12.4 mg/g of non-volatile secondary metabolites, representing a roughly 20% increase compared to the dark condition (D). This suggests that the magenta LED condition not only promotes callus proliferation but also enhances the production of secondary metabolites. Importantly, this signifies that a flavonoid increase can occur without the need for pathogen treatment, such as fungal treatment, highlighting a significant aspect revealed by this study.

### 3.3. Secondary Metabolite Profiling via HPLC

Secondary metabolites are vital compounds produced by plants in response to environmental factors and growth conditions. Edelweiss callus features specific indicators, namely leontopodic acid A and B [33]. It is crucial to confirm the augmentation of these indicators through the application of LED. To investigate the potential impact of different light conditions on the secondary metabolite profile of Edelweiss callus, we conducted HPLC analysis on extracts from callus cultures grown under varying conditions. The HPLC chromatography revealed that Edelweiss callus extracts treated with M-LED exhibited a high concentration of secondary metabolites, including leontopodic acid A (55.11 mg/g DW), leontopodic acid B (60.12 mg/g DW), and chlorogenic acid (34.01 mg/g DW) (Table 3). This underscores that M-LED illumination not only contributes to the increase in TFC and TPC but is also indispensable for the elevation of key substances in Edelweiss, such as the leontopodic acid series.

### 3.4. Comparison of Protein Concentrations

**Figure 4:**
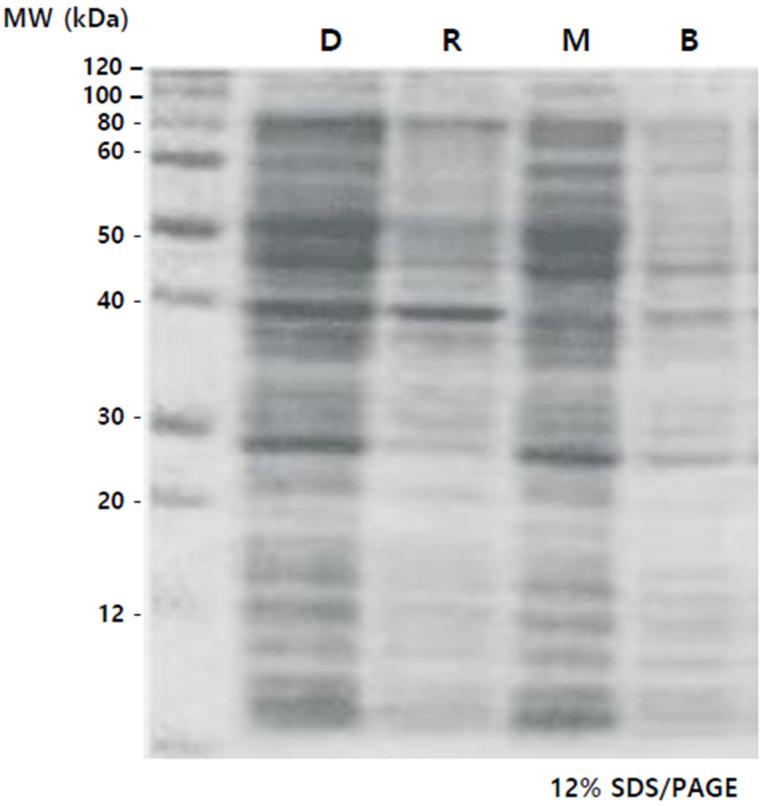
The protein profile of Edelweiss callus cultivated under diverse light sources was examined using SDS/PAGE gel. The soluble total protein from the Edelweiss callus was loaded and separated accordingly. D: dark condition, R: red light condition, B: blue light condition, M: magenta light condition.

The complete set of proteins underwent electrophoresis on a 12% SDS/PAGE gel, and the protein patterns, delineated by molecular weight, were identified as shown in Figure 4. Elevated protein concentrations were observed in Lanes 1 (D) and 3 (M) compared to Lanes 2 (R) and 4 (B), especially in the 40 to 60 kDa range. In the case of M, it was observed that the 40 kDa protein, which was prominently present in D and R, was relatively less abundant. The plant group subjected to specific wavelength LEDs witnessed a rise in protein levels. Specifically, there was a notable increase in protein content under the mixed lights, and the color combination used in this study is the same as in previous reports [40].

To determine protein concentrations in the callus under different light conditions, total soluble proteins were quantified using the BCA method, with specific concentrations for each condition: D (DARK)—411.3 μg/mL, R (RED)—239.2 μg/mL, M (MAGENTA)—439.5 μg/mL, B (BLUE)—199.4 μg/mL. The tendency of total soluble protein and the density trend of proteins through electrophoresis were similar.

### 3.5. EV Analysis from Edelweiss Callus

EVs, released by almost every cell type, play a pivotal role in intercellular communication by transporting a diverse range of cargo, including DNA, RNA, RNAi, proteins, viruses, lipids, small molecules, and soluble factors [41,42]. To investigate the potential enhancement of EV production using M-LED compared to the standard culture condition (darkness, D), we conducted an analysis employing liquid suspension culture.

We separated EVs from Edelweiss callus liquid suspension culture under both darkness (D) and M-LED conditions by passing them through 100-mesh and 300-mesh filters. After centrifugation at 3000 to 4000× *g*, only the supernatant was collected to remove cell residues. Subsequent centrifugation at 100,000 to 150,000× *g* for 2 h allowed EVs to settle, forming a pellet layer. Isolated EVs were reconstituted in sterile water and sterilized through a 0.22 µm filter. To conclude, we characterized the EVs derived from Edelweiss callus using NTA equipment (ZetaView, Particle Metrix, Inning am Ammersee, Germany) to validate their size and concentration based on the respective culture treatment conditions. This comprehensive approach provides insights into the potential impact of M-LED on EV production, offering valuable information for advancing our understanding of intercellular communication mechanisms.

**Figure 5:**
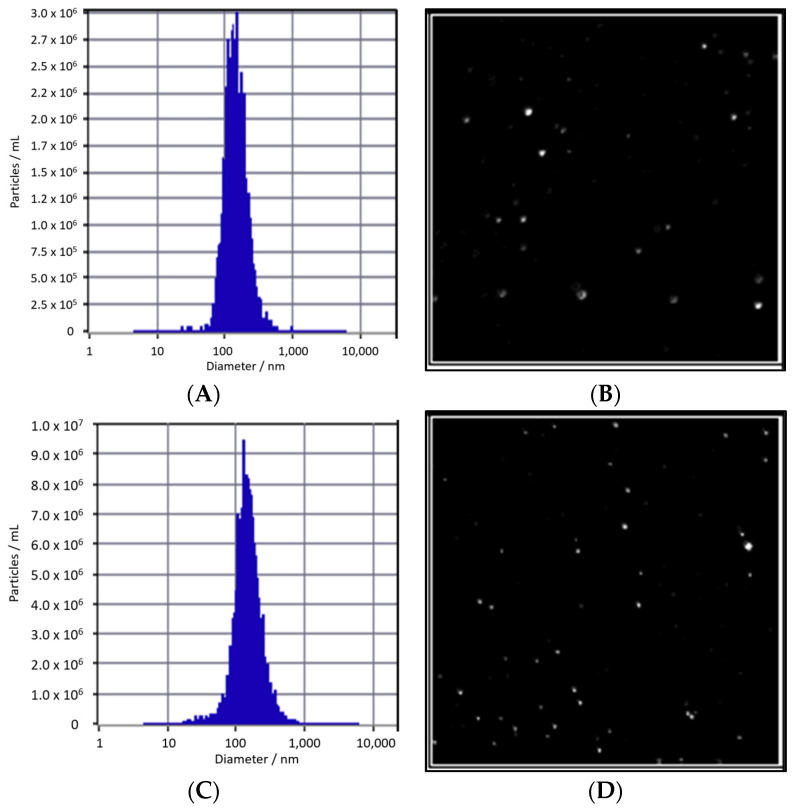
NTA analysis comparing the size (**A**) and image (**B**) of EVs derived from D-Edel-Callus (D-Edel-Callus-EV), as well as the size (**C**) and image (**D**) of EVs derived from M-Edel-Callus (M-Edel-Callus-EV). D-Edel-Callus-EV exhibits a particle size of 141.3 nm with a concentration of 1.3 × 10^11^ particles/mL, while M-Edel-Callus-EV, slightly smaller at 139.2 nm, demonstrates a higher concentration of 3.4 × 10^11^ particles/mL. This indicates a 2.3-fold increase in concentration when exposed to magenta light.

There is no notable difference in particle size between Edelweiss callus-derived extracellular vesicles (EVs) cultured under M-LED, measuring 139.2 nm, and those cultured under dark conditions, measuring 141.3 nm, as illustrated in Figure 5. However, a notable difference emerges in terms of concentration. EVs obtained from D culture exhibit a count of 1.3 × 10^11^ particles/mL, while EVs derived from M-LED culture show an approximately 2.6-fold increase, boasting a count of 3.4 × 10^11^ particles/mL. This underscores the capability of M-LED, incorporating a 50:50 ratio of blue and red LED light, to augment EV yield not only in solid culture but also during liquid suspension culture. The amplified number of EVs holds promise for elevating the concentration of diverse, active EV ingredients.

Building on these findings, it becomes evident that M-LED surpasses R-LED and B-LED in various aspects, including callus growth, proliferation, secondary metabolites, and proteins. The superior outcomes observed with M-LED suggest its potential for optimizing the production of valuable components in Edelweiss callus cultures.

### 3.6. The Increase in EV Production in Different Plant Callus Cultures Treated with LEDs

The application of M-LED led to a notable enhancement in the production of Edelweiss EVs. While reports on the amplification of EV numbers in animal cells using LEDs are scarce [43], there is a distinct lack of cases involving plant cells and LED illumination. To investigate the universality of this phenomenon, the same conditions were employed to culture *Centella asiatica* and *Panax ginseng* callus. Table 4 reveals a 2.41-fold increase in EVs derived from *Centella asiatica* and a 2.27-fold increase in *Panax ginseng*, both attributed to the magenta LED treatment. Remarkably, there were no significant differences in particle size during this process.

**Figure 6:**
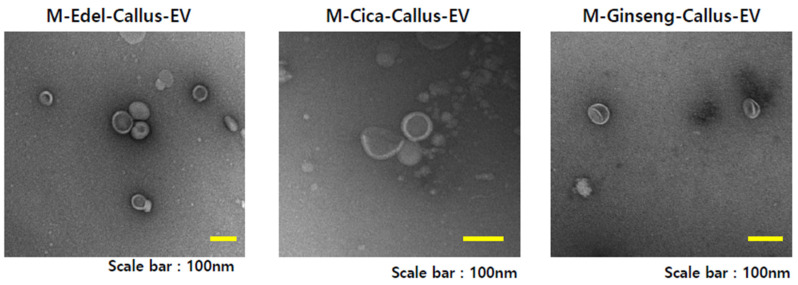
Transmission electron microscopy (TEM) image of EVs derived from Edelweiss (M-Edel-Callus-EV), *Centella asiatica* (M-Cica-Callus-EV), and *Panax ginseng* (M-Ginseng-Callus-EV) under magenta light conditions.

Similar to the Edelweiss scenario, the utilization of magenta mixed light LED, comprising an equal combination of blue and red light, resulted in an elevated EV yield during cultivation. This suggests a robust correlation with the heightened production of active compounds. Importantly, this study presents, for the first time, evidence that EVs from *Centella asiatica*, *Panax ginseng*, and Edelweiss experience an increase due to LED treatment. The findings underscore the potential of M-LED in enhancing EV production across different plant species, opening new avenues for understanding and optimizing the cultivation of valuable bioactive compounds. Figure 6 illustrates transmission electron microscopy (TEM) images capturing EVs derived from Edelweiss (M-Edel-Callus-EV), *Centella asiatica* (M-Cica-Callus-EV), and *Panax ginseng* (M-Ginseng-Callus-EV) under magenta light conditions.

### 3.7. Cell Viability Assessment

We adjusted the particle count to be the same for EVs increased by LED and those produced under dark conditions to enable an accurate comparison. To evaluate the cell-based effectiveness of Edelweiss callus EVs cultured under M-LED treatment compared to dark conditions, cell viability experiments were conducted. HaCaT cells (Human keratinocytes), Detroit cells (fibroblasts), and B16F10 cells (a mouse-derived melanoma cell line) were subjected to varying concentrations of EVs. As indicated in Supplementary Table S1, it was observed that Edelweiss CE cultured under M-LED treatment conditions exhibited a marginal reduction in cell viability of up to 10% in HaCaT cells, Detroit cells, and B16F10 cells. Importantly, these extracts demonstrated no cytotoxicity at a 5% treatment concentration, affirming their safety and non-irritating nature toward the cells.

### 3.8. Intracellular ROS Assessment

Reactive oxygen species (ROS) trigger MMPs to degrade ECM proteins, contributing to skin aging [44]. TIMPs indirectly influence skin aging by regulating MMPs. EV therapy reduces ROS, which is linked to UV-induced skin aging [12,45]. Aloe vera-derived EVs demonstrate cytocompatibility, decrease intracellular ROS, and boost antioxidant defenses in skin cells [16].

**Figure 7:**
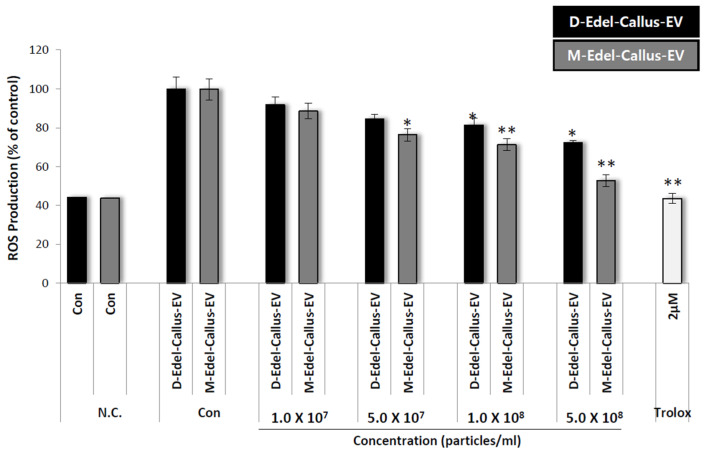
Intracellular antioxidant activity was assessed using EVs derived from Edelweiss callus, specifically designated as D-Edel-Callus-EV and M-Edel-Callus-EV. These EVs were subjected to varying concentrations in the analysis. A 2 μM Trolox solution served as the positive control. Statistical significance concerning the UV-treated control (Con) was determined (* *p* < 0.05 and ** *p* < 0.01).

In a 96-well plate, HaCaT cells were cultured and treated with CM-H2DCFDA and various concentrations, including Trolox as a positive control, followed by exposure to 500 mJ/cm^2^ of UVB. Results in Figure 7 show that Edelweiss callus EVs cultured under M-LED treatment inhibit ROS production concentration-dependently, surpassing those from callus cultured in the dark. Despite both groups having the same particle number, the difference in efficacy is likely due to the simultaneous increase in active components according to LED irradiation. It is thought that differences in substances such as leontopodic acid explain the variation in efficacy when EVs are incorporated.

### 3.9. Extracellular Melanin Content Analysis

To assess the whitening effect, an experiment was conducted using callus EVs from Edelweiss cultured under both D (dark) and M-LED (magenta light-emitting diode) treatment conditions. B16F10 melanoma cells, derived from mice, were seeded into 24-well plates at a concentration of 3 × 10^4^ cells per well using a DMEM medium. The cells were cultured for 24 h at 37 °C in a 5% CO_2_ environment. To induce melanin production, 50 nM of α-MSH was added to the cells cultured for 24 h.

Following this, each callus EV obtained from either the M-LED or D conditions was mixed with the medium at various concentrations. The mixtures were then added to the respective wells, and the cells were cultured at 37 °C with 5% CO_2_ for 72 h. After completing the culture period, the culture solutions were collected.

The amount of extracellular melanin produced during the 72 h culture was measured by taking 1 mL of the culture solution and recording the absorbance at 450 nm using an ELISA plate reader. The inhibition rate of melanin production was calculated based on the absorbance of a blank control that used DPBS (Dulbecco’s Phosphate-Buffered Saline) instead of the sample solution. A positive control, 200 µg/mL of albumin, was included.

**Figure 8:**
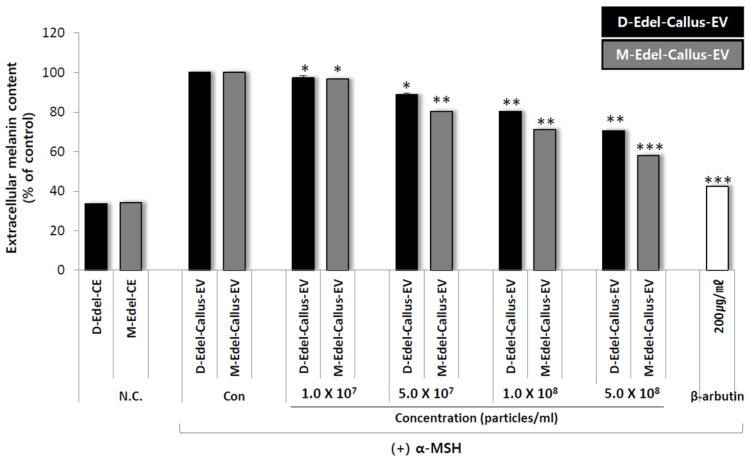
The assessment of extracellular melanin production in the mouse-derived melanoma cell line (B16F10) involved treating the cells with EVs derived from Edelweiss callus, specifically designated as D-Edel-Callus-EV and M-Edel-Callus-EV. Varying concentrations of these EVs were employed in the evaluation. Following a 24 h culture period, the cells were stimulated with α-MSH to induce melanin production. Significance in comparison to the control (Con) was determined (* *p* < 0.05, ** *p* < 0.01, and *** *p* < 0.001).

The results, as shown in Figure 8, confirm that Edelweiss callus EVs cultured under M-LED treatment conditions exhibit high activity in reducing the production of extracellular melanin. Despite having the same number of particles, the difference in efficacy between magenta and dark aligns with the earlier antioxidant results. It is suggested that more bioactive substances are produced in response to light stimulation, leading to the creation of effective EVs. LED not only increases the quantity of EVs but also enhances their quality.

### 3.10. Expression of AQP3, Filaggrin, and Collagen I Proteins

**Figure 9:**
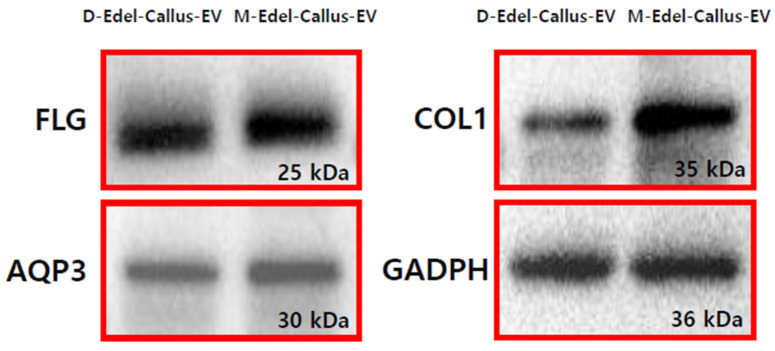
Western blot analysis images depicting the protein expression of Filaggrin, AQP3, and Collagen I are presented. Detroit cells (fibroblasts) underwent treatment with 5 × 10^8^ Particles/mL of D-Edel-Callus-EV and M-Edel-Callus-EV for 24 h. Notably, the particle numbers of EVs treated with magenta LED (M-Edel-Callus-EV) and those under dark conditions (D-Edel-Callus-EV) were identical. The proteins examined in this analysis encompass Filaggrin (filament-aggregating protein), AQP3 (aquaporin 3), and Col1 (collagen I), with GADPH (glyceraldehyde 3-phosphate dehydrogenase) employed as a control.

Filaggrin (FLG) is crucial for the skin’s protective barrier, and its deficiency is linked to conditions like atopic dermatitis [46]. Aquaporin 3 (AQP3) facilitates water and glycerol movement across cell membranes [47], while collagen (COL1) decreases with aging. In the experiment depicted in Figure 9, EVs derived from Edelweiss callus, cultured under magenta LED (M-LED) or darkness (D), were applied to fibroblasts and incubated for 24 h. Following treatment, intracellular proteins were extracted, and their concentration was measured. Subsequent steps included electrophoresis and transfer to a PVDF membrane. Antibodies targeting FLG, AQP3, and COL1 were employed to evaluate protein expression. Notably, M-Edel-CE, treated with combined light, exhibited higher expression, particularly with a more than five-fold increase in COL1, indicating a significant impact on key proteins associated with skin health. This observation aligns with previous findings related to antioxidants and melanin, suggesting that LED treatment effectively generates high-quality EVs with diverse anti-aging benefits.

## 4. Discussion

Aging and skin diseases pose a multifaceted burden, impacting mental, social, and financial aspects. Existing treatments are insufficient, prompting an urgent need for more effective therapeutic strategies through additional research [3,4]. EVs have emerged as potential treatments for skin aging [5], facilitating intercellular communication through biomolecules. Despite limited understanding, plant-derived EVs show promise in skin rejuvenation [17].

EVs derived from apples contain flavonoids and furanocoumarins known for their toxicity against pathogens (fungal species) and insects [39]. From this fact, it can be understood that EVs are generated by pathogens. Recent investigations propose a significant role of plant-released EVs in facilitating cross-border communication between plants and pathogens [48]. Both plants and pathogens release a myriad of molecules into the extracellular environment to support this essential process, which is crucial for plant defense and pathogen virulence. The noteworthy involvement of EVs stands out in achieving this communication. Consequently, pathogens are recognized as one of the primary producers of EVs. However, the term “pathogen” is perceived negatively by consumers. Moreover, concerns arise regarding the safety aspects of the production of efficacy substances, such as EVs, induced by pathogen stimulation.

The research underscores the significance of Edelweiss, a plant renowned for its medicinal properties, while emphasizing its endangered status [31]. The passage highlights a gap in research related to the use of LED technology for increasing bioactive substances and enhancing EV production in Edelweiss. While there are existing reports on the simple callus culture and component analysis of Edelweiss, specific investigations of LED’s impact on bioactive substances and EV production are lacking [33]. The study being referenced focuses on *Panax vietnamensis* and observes that applying specific wavelengths of LED light significantly improves callus growth and influences the production of ginsenosides under certain LED conditions [25]. The objective is to optimize LED conditions for Edelweiss callus, evaluating growth indicators and investigating the increase in EVs. In contrast to earlier investigations on Edelweiss callus, this study prioritizes the role of LED exposure and EV production. To enhance EV production, this study explores the impact of LEDs, focusing specifically on Edelweiss callus culture.

The investigation into the impact of red (R), blue (B), and magenta (M) LEDs on Edelweiss callus growth and EV production reveals compelling results. When comparing only blue and red, it can be observed that blue is superior in terms of TFC and TPC. The report aligns with findings that indicate an increase in flavonoids and antioxidants during plant cultivation under blue light exposure [49]. The involvement of blue LED light in promoting the elevation of active substances is suggested, as evidenced by its recommendation for enhancing the content of phenolic compounds [50]. Blue light has its drawbacks, one of which is the inhibition of growth. For instance, there are reports indicating that blue light suppresses the generation of stems [51]. In contrast, red light serves as a supportive means of promoting growth. Red light facilitates both root growth and the accumulation of plant organic matter [52]. This leads to the conclusion that red light influences the dry weight of plants. Magenta, created by the combination of blue and red with their distinct properties, undoubtedly possesses unique advantages. Magenta LED conditions significantly enhance callus growth in terms of both fresh and dry weight compared to red and blue LEDs.

This suggests that magenta LEDs contribute to increased callus proliferation, showcasing a growth rate comparable to the dark condition. The study highlights a higher fresh callus weight under magenta light, emphasizing its potential for optimizing callus biomass. These findings align with the following observations: blue light enhances photosynthesis and decreases leaf area, while red light encourages the development of larger leaf areas and longer roots [50]. Although there are differences between plants and calluses, the overall trends are consistent.

The research explores the influence of LED conditions on secondary metabolites, specifically total flavonoid content (TFC) and total phenolic content (TPC). Magenta LED conditions yield the highest TFC and TPC values, indicating an approximate 20% increase in non-volatile secondary metabolites compared to the dark condition. This suggests that magenta LED not only promotes callus proliferation but also enhances the production of key secondary metabolites, including flavonoids. It appears that red stimulates callus proliferation, while blue enhances the production of secondary metabolites.

High-performance liquid chromatography (HPLC) analysis confirms the elevated concentration of specific secondary metabolites, including leontopodic acid A and B, under magenta LED conditions. This underscores the importance of magenta LED illumination in augmenting key substances in Edelweiss, contributing to its potential applications in cosmetics. The study observes an increase in protein concentrations, particularly in the 40 to 60 kDa range, under magenta LED conditions. This rise in protein levels is notable when compared to red and blue LED conditions, highlighting the influence of specific wavelength LEDs, especially magenta, on protein content in Edelweiss callus.

The most significant finding of the study is the notable increase in EV production under magenta LED conditions. While the particle size of EVs remains consistent, there is a significant increase in concentration under magenta LED, indicating its potential to enhance the yield of active EV ingredients. This outcome is consistent across different plant species (*Centella asiatica* and *Panax ginseng*), suggesting the universality of this phenomenon. The fact that EVs can be generated by specific light from LEDs in this study provides insight into the ability to produce EVs without using pathogens. Notably, while there is a documented rise in light-induced EVs in marine bacteria [53], there is presently no observed increase in EVs in plants because of exposure to LED light. Our report is considered the first to document an increase in EV in plant callus due to specific LED exposure.

Cell viability experiments reveal that Edelweiss callus EVs cultured under magenta LED conditions exhibit a marginal reduction in cell viability, indicating their safety and non-irritating nature. Additionally, magenta LED-cultured EVs demonstrate an inhibitory effect on reactive oxygen species (ROS) production, contributing to potential anti-aging benefits. The study demonstrates the ability of Edelweiss callus EVs cultured under magenta LED conditions to effectively reduce extracellular melanin production in B16F10 melanoma cells. This aligns with antioxidant results and suggests that magenta LED treatment leads to the creation of effective EVs with diverse anti-aging benefits.

Magenta LED-cultured Edelweiss callus-derived EVs show higher expression of key proteins associated with skin health, including Filaggrin (FLG), Aquaporin 3 (AQP3), and Collagen I (COL1). This indicates a significant impact on proteins crucial for the skin’s protective barrier, water movement, and collagen production, reinforcing the potential anti-aging benefits of magenta LED treatment. The unique contribution lies in applying magenta LED to Edelweiss callus, demonstrating a significant rise in EV production, potentially enhancing skin-related attributes. EVs generated from plant organisms treated with magenta light indicate a superior quality compared to those produced under normal conditions. This implies that the quality and quantity of EVs have been enhanced by the respective properties of blue and red light. Extracellular vesicles generated by magenta not only enhance quantity but also improve quality. This can be inferred from reports indicating that *Salvia miltiorrhiza* Bunge, influenced by a combination of blue and red light, not only promotes plant growth but also leads to an increase in phenolic acid contents [50]. The presence of extracellular vesicles suggests the augmentation of specific components, such as phenolic acids. Despite existing LED studies for various plants, limited attention has been given to callus cultures. The study identifies a knowledge gap regarding the increase in plant EVs due to LED exposure.

In conclusion, the study suggests that magenta LED conditions, combining blue and red LED light, hold promise for optimizing Edelweiss callus cultures by promoting callus growth, enhancing the production of secondary metabolites and proteins, and notably increasing EV yield. The findings open new avenues for understanding and optimizing the cultivation of valuable bioactive compounds in different plant species.

## Figures and Tables

**Table 1:** Comparison of callus growth rate under four different light treatments.

Contents	Dark (D)	Red (R)	Magenta (M)	Blue (B)
Fresh callus weight (FW (g)/Petri dish)	8.0 ± 1.1	4.00 ± 0.6	8.76 ± 1.6	4.5 ± 0.7
Dried callus weight (DW (g)/Petri dish)	0.31 ± 0.2	0.12 ± 0.1	0.36 ± 0.1	0.15 ± 0.1
DW/FW ratio (%)	3.88	3	4.11	3.33

**Table 2:** Comparison of total polyphenol and flavonoid contents in callus extracts cultured under varying LED light conditions. (aCE: catechin equivalents, bGAE: gallic acid equivalents, cDW: dry weight).

Contents	Dark (D)	Red (R)	Magenta (M)	Blue (B)
Total Flavonoid Content (TFC) (mg aCE/g DW)	124.8 ± 5.1	122.1 ± 4.5	143.5 ± 5.2	124.8 ± 4.2
Total Phenolic Content (TPC) (mg bGAE/g DW)	156.2 ± 6.7	136.2 ± 4.2	162.4 ± 7.2	160.2 ± 2.2
Total amount (mg/g cDW)	281.0 ± 11.8	258.3 ± 8.7	305.9 ± 12.4	285.0 ± 6.4

**Table 3:** Quantitative analysis of primary components in the callus extract (CE), which comprises 1% dried callus extracts using a hot-water method.

Compound	Edelweiss Callus Extract (CE) (mg/g DW)			
	Dark (D)	Red (R)	Magenta (M)	Blue (B)
Leontopodic acid A	45.15	35.12	55.11	46.11
Leontopodic acid B	56.2	41.45	60.12	52.14
Chlorogenic acid	25.5	24.22	34.01	24.5
Total	144.97	119.9	164.68	141.96

**Table 4:** NTA analysis was performed on *Centella asiatica* and *Panax ginseng* treated with dark or magenta light conditions. A comparison between magenta and dark conditions reveals that the particle count for *Centella asiatica* and *Panax ginseng* increased by 2.41 and 2.27 times, respectively.

Plant Species	Type of Light	Sample	Particle Size (nm)	Concentration (Particles/mL)	Increase Rate (fold)
*Centella asiatica*	D (Dark)	D-Cica-Callus-EV	141.3	1.2 × 10^11^	2.41
	M (Magenta)	M-Cica-Callus-EV	140.9	2.9 × 10^11^	
*Panax ginseng*	D (Dark)	D-Ginseng-Callus-EV	154.6	1.8 × 10^10^	2.27
	M (Magenta)	M-Ginseng-Callus-EV	150.2	4.1 × 10^10^	

## Data Availability

The data that support the findings of this study are available from the corresponding author upon reasonable request.

## Supplementary Materials

The following supporting information can be downloaded at: https://www.mdpi.com/article/10.3390/cimb45120634/s1, Table S1: Evaluation of cell toxicity was conducted, specifically focusing on particle counts of EVs subjected to magenta LED treatment (M-Edel-CE) and those kept in dark conditions (D-Edel-CE). The standardization was set at 10^9^ particles/mL, calculated from the original solution. The cell lines utilized in the study included HaCaT cells (human keratinocytes), Detroit cells (fibroblasts), and the B16F10 melanoma cell line derived from mice.
