# Supplementary material for: Improvement in Yield of Extracellular Vesicles Derived from Edelweiss Callus Treated with LED Light and Enhancement of Skin Anti-Aging Indicators
Source: Curr Issues Mol Biol. 2023 Dec 16;45(12):10159–78. doi: 10.3390/cimb45120634 (PMC10742862; doi:10.3390/cimb45120634)
Supplement: Supplementary file 1 [file cimb-45-00634-s001.zip › cimb-2763705-supplementary.pdf]

Supplementary Table S1. Evaluation of cell toxicity was conducted, specifically focusing on particle counts of EVs subjected to magenta LED treatment (M-Edel-CE) and those kept in dark conditions (D-Edel-CE). The standardization was set at 10^9 particles/mL, calculated from the original solution. The cell lines utilized in the study included HaCaT cells (human keratinocytes), Detroit cells (fibroblasts), and the B16F10 melanoma cell line derived from mice.

Treatment	Treated Concentration (%)	HaCaT Cell Viability (%)	Detroit Cell Viability (%)	B16F10 Cell Viability (%)
Control	-	100	100	100
D-Edel-CE	0.1	100	100	100
	0.5	98	100	102
	1	102	98	98
	5	101	105	102
	10	98	95	97
M-Edel-CE	0.1	100	100	100
	0.5	102	101	100
	1	102	102	102
	5	111	110	112
	10	95	95	92
